# Development and efficacy assessment of hand sanitizers and polylactic acid films incorporating caffeic acid and vanillin for enhanced antiviral properties against HCoV-229E

**DOI:** 10.1186/s12985-023-02159-z

**Published:** 2023-08-28

**Authors:** Seok-Woo Hyun, Sangha Han, Jeong Won Son, Min Su Song, Dan Ah Kim, Sang-Do Ha

**Affiliations:** Department of Food Science and Technology, Advanced Food Safety Research Group, Chung-Ang University, Anseong-si, Gyeonggi-do 17546 Republic of Korea

**Keywords:** SARS-CoV-2, Hand sanitizers, Poly lactic acid, Caffeic acid, Vanillin

## Abstract

**Background:**

Although three years after the outbreak of SARS-CoV-2, the virus is still having a significant impact on human health and the global economy. Infection through respiratory droplets is the main transmission route, but the transmission of the virus by surface contact cannot be ignored. Hand sanitizers and antiviral films can be applied to control SARS-CoV-2, but sanitizers and films show drawbacks such as resistance of the virus against ethanol and environmental problems including the overuse of plastics. Therefore, this study suggested applying natural substrates to hand sanitizers and antiviral films made of biodegradable plastic (PLA). This approach is expected to provide advantages for the easy control of SARS-CoV-2 through the application of natural substances.

**Methods:**

Antiviral disinfectants and films were manufactured by adding caffeic acid and vanillin to ethanol, isopropyl alcohol, benzalkonium chloride, and PLA. Antiviral efficacies were evaluated with slightly modified international standard testing methods EN 14,476 and ISO 21,702.

**Results:**

In suspension, all the hand sanitizers evaluated in this study showed a reduction of more than 4 log within 2 min against HCoV-229E. After natural substances were added to the hand sanitizers, the time needed to reach the detection limit of the viral titer was shortened both in suspension and porcine skin. However, no difference in the time needed to reach the detection limit of the viral titer was observed in benzalkonium chloride. In the case of antiviral films, those made using both PLA and natural substances showed a 1 log reduction of HCoV-229E compared to the neat PLA film for all treatment groups. Furthermore, the influence of the organic load was evaluated according to the number of contacts of the antiviral products with porcine skin. Ten rubs on the skin resulted in slightly higher antiviral activity than 50 rubs.

**Conclusion:**

This study revealed that caffeic acid and vanillin can be effectively used to control HCoV-229E for hand sanitizers and antiviral films. In addition, it is recommended to remove organic matter from the skin for maintaining the antiviral activity of hand sanitizer and antiviral film as the antiviral activity decreased as the organic load increased in this study.

## Introduction

Severe acute respiratory syndrome coronavirus 2 (SARS-CoV-2) first emerged in Wuhan, China, in 2019 and spread through the world in a short period, causing about 645 million infections and 6.6 million deaths as of November 2022 [1]. SARS-CoV-2 is classified as a new beta-coronavirus in the family Coronaviridae. SARS-CoV-2 is an enveloped virus with positive sense, single-strand RNA [2]. This virus not only causes respiratory symptoms like coughing, a sore throat, difficulty of breathing, and a runny nose, but also produces gastrointestinal symptoms such as diarrhea, vomiting, and abdominal pain [3]. According to the Centers for Disease Control and Prevention (CDC) of the United States, the main transmission route for SARS-CoV-2 is the exposure of the mucous membrane to contaminated surfaces through the hands or direct exposure of the respiratory tract to droplets containing the virus through person-to-person contact [4]. For these reasons, the World Health Organization (WHO) and the CDC have consistently emphasized the importance of hand hygiene [5] and have requested that common contact surfaces such as stainless steel, plastic, and glass, which show high stability for SARS-CoV-2, are kept in a clean condition [6, 7]. In particular, the CDC has selected shopping cart handles, elevator buttons, keyboards, and faucets as examples of surfaces that people routinely contact [8]. Therefore, many studies have been conducted to prevent secondary contagion by using an antiviral film as an additional method for disinfecting these surfaces [9].

Antiviral films incorporating copper and silver are currently commercialized and are widely used [10]. The antiviral properties of these films could be strengthened by adding ingredients such as tea extract and essential oils [11]. Additional research has been conducted to minimize the exposure of people to virus particles and prevent these particles from settling on the surface by applying physical properties such as super-hydrophobicity [10]. Hence, PLA was selected because it can be conjugated with other materials and inhibit the adhesion of a virus on a surface through hydrophobicity [12]. Furthermore, PLA can be used in various fields such as medicine, packaging, electronics, automobiles, and textiles due to its excellent characteristics including eco-friendliness, biodegradability, biocompatibility, and hydrophobicity [12].

The efficiency of hand sanitizers has been evaluated since the SARS-CoV-2 outbreak. Research by Herdt and co-workers [13] indicates a reduction above 4 log during 30 s of treatment with ethanol and quaternary ammonium-based hand sanitizers. In addition, research was also performed to evaluate the antiviral activity by adjusting the proportion of disinfectants [14] or adding natural substances with antiviral effects to the existing formulations to increase the virucidal power [15, 16]. Natural substances have provided outstanding treatment alternatives for various infectious diseases since ancient times [17]. In particular, phytochemicals such as flavonoids, alkaloids, and polyphenols, which are mostly found in plants, provide benefits to humans [18]. Furthermore, phytochemicals show antimicrobial activity [19], especially alkaloids, terpenes, flavonoids, and glycosides show strong antiviral activity against highly pathogenic influenza, dengue, polio, and adenoviruses [20].

Caffeic acid is a polyphenol contained in coffee, berries, and tea [21]. Caffeic acid shows biological activities including anti-inflammatory, anti-cancer, and immune enhancement effects [22, 23], and can play a role as an antiviral substance. Several studies have reported that caffeic acid is effective against the virus that causes severe fever thrombocytopenia syndrome as it inhibits the binding of the virus to a host cell and disrupts the replication of the viral RNA of herpes simplex and the influenza A virus [24–26]. The antiviral mechanisms of caffeic acid against SARS-CoV-2 are considered to be due to its targeting of structures of the virus, spike protein, non-structured protein, and main protease [21]. Although the antiviral mechanism has not been clearly identified, a study [27] mentioned that caffeic acid is attached to the cell-surface heat shock protein A5 domain, using a high binding affinity, which functions as the mechanism behind the SARS-CoV-2 recognition of the host, thereby suppressing the binding between host and virus. In addition, other studies [28, 29] mentioned that caffeic acid can be attached to the main protease and non-structured protein, which are essential for the maturity and replication of the virus, thereby inhibiting the proteolytic process and reducing enzymatic activity by competitive inhibition, in turn reducing the possibility of SARS-CoV-2 transmission.

Vanillin is a phenolic aldehyde isolated from the vanilla bean and is mainly used in aroma substances, food additives, perfumes, and pharmaceutical products [30]. Recently, several studies on the health benefits of vanillin including anticancer, antioxidant, anti-inflammatory [31–33], and antiviral effects [34] have been published. Studies [35, 36] have proven the effectiveness of vanillin against the herpes simplex virus types 1 and 2 and the H1N1 virus. A 60% reduction of the cytopathic effect was achieved using 500 ppm of *M. officinalis* extract, whereas an 85% reduction of the cytopathic effect was achieved using 125 ppm of vanillin. Similar to caffeic acid, the exact mechanism behind the action of vanillin has not been identified, but vanillin targets the main protease and spike protein of SARS-CoV-2 [21]. Vanillin can maintain a stable binding state to the protein of SARS-CoV-2 through van der Waals, hydrogen bonds, and hydrophobic bonds, which can reduce host infectivity by the competitive connection between the receptor-binding domain of SARS-CoV-2 and angiotensin-converting enzyme 2 in the spike protein [34, 37]. Moreover, this may be a way to inhibit the translation of viral polyproteins and replicase activity in the main protease [38].

Hand sanitizers and antiviral films are considered to be effective strategies for reducing the spread of SARS-CoV-2. However, their antiviral activity could be limited in environments where organic substances and envelope viruses exist [39]. In addition, due to the continuous application of hand sanitizers or antiviral films, pathogens develop resistance to the active ingredients, which is a serious problem [40]. A recent study comparing the viability of the Omicron and Wuhan strains in plastic and skin showed that the viability of Omicron was more than twice that of Wuhan, and a 7% higher ethanol concentration was needed in disinfectant experiments to achieve the same inactivation compared to the Wuhan strain [41]. Therefore, developing safe and effective hand sanitizers and antiviral films that can create synergistic effects to inactivate SARS-CoV-2 is necessary.

The main purpose of this study was to evaluate the antiviral effect of caffeic acid and vanillin as natural substances against HCoV-229E, a surrogate of SARS-CoV-2. Novel hand sanitizers and PLA films that contained vanillin and caffeic acid were manufactured to evaluate their antiviral properties. The antiviral effects of the newly formulated hand sanitizers and antiviral PLA films were evaluated using a porcine skin model as a surrogate of human skin.

## Materials & methods

### Cell line preparation

Human lung fibroblast MRC-5 cells (Medical Research Council cell strain 5) were purchased from the American Type Culture Collection (ATCC; Rockville, MD, USA). MRC-5 cells were cultured using Eagle’s minimum essential medium (MEM; Sigma-Aldrich, St. Louis, MO, USA) mixed with 10% fetal bovine serum (FBS; Gibco, Rockville, MD, USA) and 1% penicillin-streptomycin (Gibco). Cells were cultured in a 75 cm^2^ culture flask and incubated at 37℃ with 5% CO_2_. If the density of cells was nearly 100% under a microscope, sub-culture proceeded. First, the cell culture media was discarded and washed twice with Dulbecco’s phosphate-buffered saline (DPBS; Sigma-Aldrich) solution. Then, the PBS solution was aspirated and 1 mL of 0.25% trypsin-EDTA(1X) (Gibco) was gently added to detach the cells from the bottom of the flask. After 6 to 10 min of incubation at 37℃ with 5% CO_2_, cells were centrifuged at 300 ×g for 5 min. The cell pellet was gently mixed with 1 mL of fresh cell culture media and transferred into a new flask. All these procedures were performed every 2 to 4 days.

### Virus preparation

Human coronavirus 229E (HCoV-229E) was obtained from the American Type Culture Collection (ATCC; Rockville, MD, USA) for use as a surrogate of SARS-CoV-2. When the density of the cells was nearly 90–100% under a microscope, the cell culture media was discarded and washed with DPBS twice. After DPBS was aspirated, the exact amount of the HCoV-229E suspension was calculated with MOI (multiplicity of infection) = 0.1 and the virus was gently injected into the cell culture flask. Then, the flask was placed in an incubator with 5% CO_2_ at 33℃ for 2 h to ensure virus attachment. During this time, the flask was taken from the incubator and gently shaken every 30 min. At the end of these procedures, the maintenance media (MEM with 1% FBS) was prepared and added until 10 mL of total volume was reached. The CPE (cytopathic effect) was observed for 3 to 7 days, and if the CPE was confirmed, the sample was stored deep-freezer to perform three freeze-thaw cycles. All HCoV-229E virus suspensions were collected in 50 mL of a conical tube and centrifuged at 4000 ×g and 4℃ for 10 min. Finally, the supernatant was filtered with a 0.2 μm syringe filter to remove the cell pellet and stored deep-freezer at -80℃ before use in the experiments.

### MTT assay

The MTT [3-(4,5-dimethylthiazol-2-yl)-2,5-diphenyltetrazolium bromide] assay was performed to determine the cell viability, using caffeic acid and vanillin. MRC-5 cells were seeded with a density of 1 × 10^4^ cells/100 µL in a 96-well plate, and after 24 h, the old media was removed and replaced with new cell culture media mixed with various concentrations (30, 40, 50, 60, 70, 80, 90, and 100 µmol) of natural substances. The MTT reagent was added every 3 h before each experiment, shaken smoothly for 15 min, and incubated at 37℃ with 5% CO_2_ to generate formazan. After taking out the well plate at the exact experimental time, the media was removed, 100 µL of DMSO was added to each well, and the formazan was dissolved by pipetting at an appropriate speed. Optical density measurement was performed using a spectrophotometer (Spectra Max 190, Sunnyvale, USA) at 460 nm. The percent of cell viability was calculated using the following formula:

Cell viability (%) = [Numerical OD value – Blank value / Control OD value – Blank value] × 100.

### Preparation of commercial hand sanitizer and evaluation

A 70% ethanol-based product (AMOREPACIFIC CO., LTD, Seoul, Korea) and a 70% isopropyl alcohol-based product (Green Pharmaceutical., LTD, Seoul, Korea) were purchased. A 10% benzalkonium chloride-based product (Green Pharmaceutical., LTD) was purchased and diluted to a 0.066% concentration.

All of these hand sanitizers were tested for the virucidal activity against HCoV-229E as a surrogate of SARS-CoV-2, using a slightly modified protocol of the European Norm EN14476 to quantify the reduction value in suspension. First, the commercial hand sanitizer was vortexed for 30 s and an 800 µL aliquot was transferred to a 1.5 mL EP tube. Then, 100 µL of 0.3 g/L bovine serum albumin (BSA) was slowly added to 100 µL of virus suspension to adjust the final volume to 1 mL, and the mixture was treated with the hand sanitizer for 0.5, 1, 2, 3, and 5 min. During this treatment time, the solutions were mixed vigorously by pipetting. When each treatment time was completed, a 100 µL aliquot was taken and mixed with 900 µL of MEM + 1% FBS to neutralize the test substances. Finally, a serial dilution was performed, and the virus was inoculated to calculate the viral titer.

### Hand sanitizer formulation and evaluation

Formulated hand sanitizers were prepared by applying slightly modified WHO homemade hand sanitizer protocols (Table 1). Alcohol (99.9%) (DAEJUNG CO., LTD, Siheung, Korea), isopropyl alcohol (99.5%) (JUNSEI CO., LTD, Japan), and benzalkonium chloride (10%) (Green Pharmaceutical., LTD, Seoul, Korea) were purchased and directly mixed with glycerol, propylene glycol (Sigma-Aldrich, St. Louis, MO, USA, ACS reagent, 99.5%), and autoclaved distilled water. The pH of the three hand sanitizers were adjusted by adding 1 M of sodium hydroxide and 1 N of hydrochloric acid. Finally, all of the hand sanitizers were filtrated through a 0.22 μm filter fitted to a 250 mL storage bottle system to prevent contamination and remove debris.

Caffeic acid (Sigma-Aldrich, 98.0% HPLC) and vanillin (Sigma-Aldrich, ReagentPlus®, 99%) were used as antiviral substances. Ethanol (100 mL) was used as a solvent. Caffeic acid (108.1 mg) and vanillin (152.15 mg) were dissolved in ethanol to prepare 6000 and 10,000 µmol/mL solutions. Finally, 1 mL of the prepared solution was added to the formulated hand sanitizer to adjust the total volume to 100 mL. The experimental method is the same as mentioned in preparation of commercial hand sanitizer.

**Table 1:** Composition of the modified CAU (Chung-Ang University)-formulated hand sanitizers

Components	Ethanol-based (99.9%)	Isopropyl alcohol-based (99.5%)	Benzalkonium chloride-based (10%)
pH	7.0	7.0	6.8
(Disinfectant)	70.1 mL	70.1 mL	0.66 mL
Glycerol (98%)	0.74 mL	0.74 mL	0.74 mL
Propylene glycol (99.5%)	0.729 mL	0.729 mL	0.729 mL
Caffeic acid (6000 µmol)	1 mL	1 mL	1 mL
Vanillin (10,000 µmol)	1 mL	1 mL	1 mL
Distilled water	27.431 mL	27.431 mL	96.871 mL
Total amount	100 mL		

### Evaluation of the efficacy of the formulated hand sanitizer against the HCoV-229E suspension in a porcine skin model

Fresh swine skin was purchased from a local market in Anseong, Korea. The porcine skin was washed twice with tap water and distilled water. The porcine skin was then dried slightly and cut into 2 × 2 cm squares with a sterilized knife. Afterward, we placed the porcine skin pieces in sterile Petri dishes and irradiated them with UV light for 15 min on the epidermal and dermal sides.

The samples were transferred to a 6-well plate for smooth inoculation. HCoV-229E virus suspension (100 µL, 7.0 log TCID_50_/mL) was spot-inoculated on the epidermal side and the suspension was spread using a pipette tip for virus adhesion. After 30 min of incubation, 800 µL of the formulated hand sanitizer and 100 µL of BSA were applied to each sample to adjust the total volume to 1 mL. Each treatment was performed for 0.5, 1, 2, 3, and 5 min and samples were immediately taken out to soak in 2 mL of 1% FBS + MEM solution to neutralize the hand sanitizer. The virus was recovered by vortexing for 1 min. Finally, the sample was filtered using a 0.2 μm syringe filter and the recovered suspension was inoculated to measure the viral titer.

### Preparation of antiviral films using PLA and natural substances

Polylactic acid (PLA) (Goodfellow., LTD, England) was used as a primary coating substance. Chloroform (Sigma Aldrich, 99.5%) was used as a solvent as it yielded the highest solubility. A magnetic bar was put into a 500 mL beaker, covered with aluminum foil, and autoclaved at 121℃ for 15 min. PLA granules were mixed with chloroform for 1 to 2 h to reach a concentration of 7.6% (w/v). Mixing was stopped when it was visually confirmed that the PLA particles were completely melted.

Caffeic acid and vanillin were used as antiviral substances. Caffeic acid (108.1 mg) and vanillin (152.15 mg) were dissolved in 10 mL of ethanol to prepare 6,000 and 10,000 µmol/mL solutions. Afterward, a serial dilution was performed to prepare the corresponding 600 and 1000 µmol/mL solutions. Finally, 10 mL of this solution was mixed with 90 mL of the PLA-coating solution to adjust the total volume to 100 mL, stirred vigorously for 1 to 2 h, and 10 mL was poured into stainless steel square frames. The samples were dried at 50 to 60℃ for 2 to 3 days to attain thermodynamic properties.

### Evaluation of the efficacy of the antiviral PLA film against the HCoV-229E suspension

The efficacy of the antiviral film was evaluated using the method of Butot and co-workers [9], with slight modifications based on ISO 21,702 to measure the antiviral effects on plastic and non-porous surfaces. The coating was cut into 2 × 2 squares, using sterilized scissors, and immersed in 70% ethanol for 5 min to remove residues. Then, Whatman No. 4 filter paper was placed on a Petri dish, three films were placed on top of each other, and the films were dried back and forth for 15 min. Subsequently, 25 µL of HCoV-229E virus suspension was inoculated on each coating and dried for 30 min to ensure virus adhesion. Then, neat PLA (NP), 60 µmol caffeic acid added PLA (CP) and 100 µmol vanillin added PLA (VP) film samples were tested against HCoV-229E for 2 h at intervals of 30 min.

As soon as each processing time was over, samples were soaked in 1 mL of 1% FBS + MEM solution and the virus was recovered by vortexing for 1 min. Finally, the recovered suspension was inoculated to measure the viral titer.

### Evaluation of the efficacy of the antiviral PLA film against HCoV-229E on porcine skin according to the number of contacts

The methods for preparing the porcine skin and film samples were the same as in evaluation the efficacy of hand sanitizer in porcine skin and antiviral PLA film against HCoV-229E suspension, respectively. After placing each of the three antiviral films and porcine skin samples on a Petri dish, 25 µL of HCoV-229E suspension was inoculated on the epidermis layer of the skin. Then, the virus-inoculated epidermis was carefully picked up with sterilized forceps and rubbed with each prepared film 10 and 50 times at intervals of 30 min for 2 h. Here, 50 rubs represent the maximum number of contacts of daily average on highly touched surfaces, and 10 rubs represent a low contact frequency.

After the number of rubs was completed (for every 30 min), the sample was immediately soaked in 1 mL of 1% FBS + MEM solution, and then the virus was recovered by vortexing for 1 min. Finally, the sample was filtered with a 0.2 μm syringe filter and the recovered suspension was inoculated to measure the viral titer.

### Attenuated total reflectance-fourier transform infrared spectroscopy (ATR-FTIR) analysis

ATR-FTIR analysis was performed to verify the conjugation of the antiviral vanillin (VP)- and caffeic acid-coated (CP) films on the PLA surfaces. The surfaces were prepared in the same manner as mentioned in preparation of antiviral film using PLA and natural substances. The spectra were measured using a Nicolet iS10 FT-IR spectrometer (Thermo Fisher Scientific, Waltham, MA, USA) between 4000 and 400 cm^− 1^ wavelengths with no additional sample preparation. Different coated samples were analyzed at five random spots with 0.5 cm^− 1^ of peak-to-peak resolution per spectrum. For each spectrum, 32 scans with a peak-to-peak resolution of 4 cm^− 1^ were combined. After treatment, data were recorded by 64-bit high-performance FT-IR software. Documented data were aligned and the wavenumbers of the major spectra were detected by OMNIC™ Spectra Software (Thermo Fisher Scientific) in transmission (%T) mode [42].

### TCID_50_ assay

In all experiments, the HCoV-229E titer was calculated using the tissue culture infectious dose assay. Briefly, MRC-5 cells were seeded with a density of 1 × 10^4^ cells/100 µL in a 96-well plate and incubated at 37℃ with 5% CO_2_ until 50% density was observed under a microscope. After 24 h, the cell culture media was discarded and washed with DPBS once. The virus sample was prepared with a 10-fold serial dilution, using 1% FBS of MEM cell culture media, and inoculated. The inoculated 96-well plate was incubated at 33℃ with 5% CO_2_ for 5 days to determine the CPE with a microscope. The CPE was calculated using the Reed-Muench method (also known as the difference of logarithms):

Difference of logarithms = [(mortality at a dilution just above 50%)-50%] / [(mortality just above 50%) -(mortality just below 50%)]

### Statistical analysis

Experiments were conducted independently in triplicate, using at least three samples. Expressions of viral titer were calculated as logarithmic functions (log TCID_50_/mL in suspension and porcine skin). All data were stated as mean ± standard deviation (SD). IBM SPSS Statistics version 26 (IBM Corp, Armonk, NY, USA) was used to perform Duncan’s multiple range test and one-way analysis of variance (ANOVA). Graphs were prepared using Sigma-Plot version 10.0 (Systat Software, Inc., San Jose, CA, USA). Significant differences between the treatment time of the hand sanitizer and the film were determined using *p* < 0.05.

## Results

### Evaluation of the efficacy of commercial hand sanitizers against the HCoV-229E suspension

**Fig. 1:**
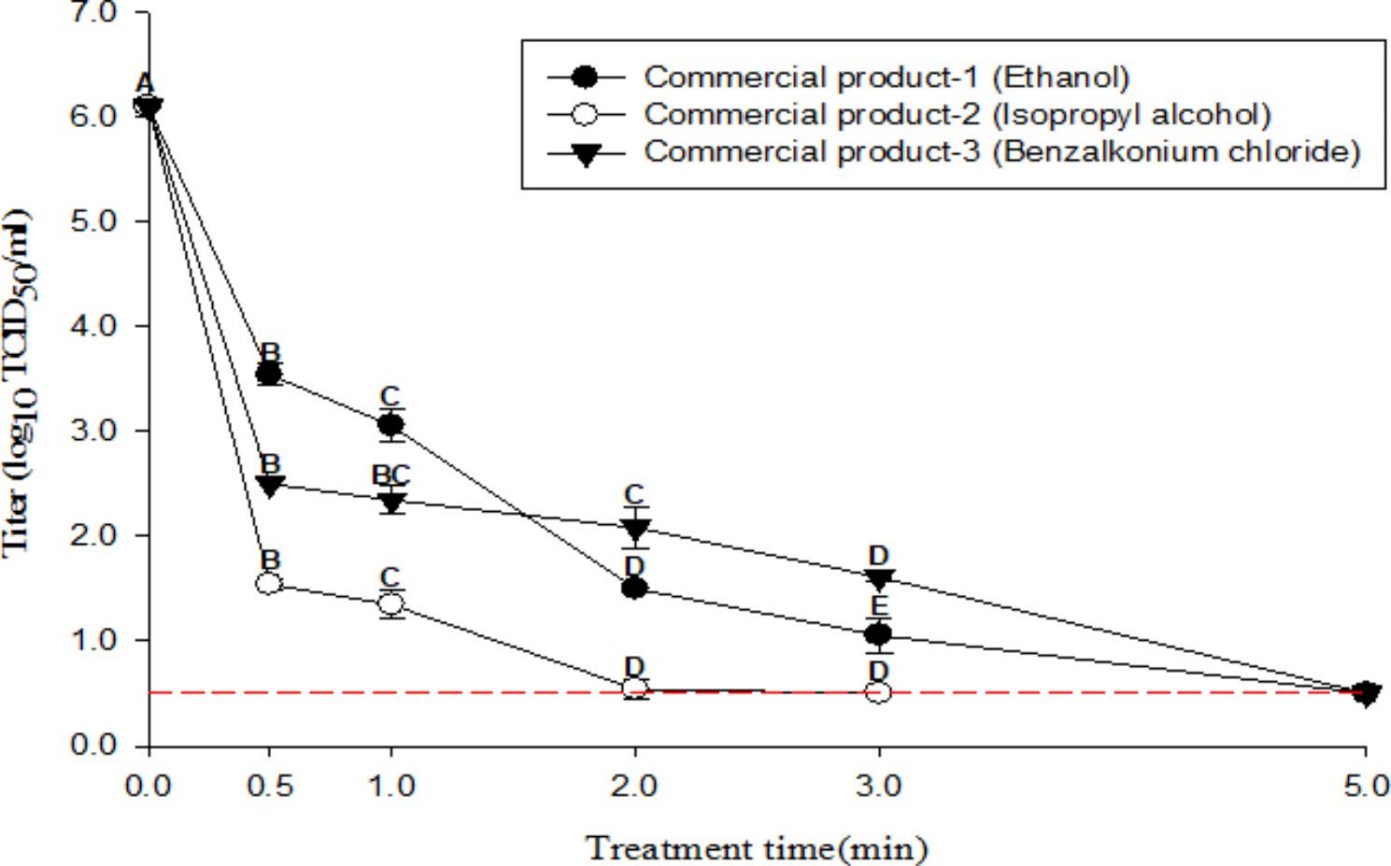
Inactivation of HCoV-229E in suspension by commercial hand sanitizers. The error bars represent the standard deviations of the means (SD). A-E indicate significant differences (p < 0.05) by treatment time. The short dash line indicates the detection limit (0.5 log TCID_50_/mL)

Figure 1 shows the efficacy of ethanol-, isopropyl alcohol-, and benzalkonium chloride-based commercial hand sanitizers against HCoV-229E in suspension. The initial titer of HCoV-229E was 6.1 log TCID_50_/mL. The commercial 70% ethanol sanitizer showed HCoV-229E log reduction values of 2.5, 3.0, 4.6, and 5.0 log_10_ TCID_50_/mL for 0.5, 1, 2, and 3 min of treatment, and no CPE (detection limit: 0.5 log TCID_50_/mL) was observed at 5 min of treatment, respectively.

The 70% isopropyl alcohol sanitizer showed log reduction values of 4.5, 4.7, and 5.5 for 0.5, 1, and 2 min, and no CPE was observed (detection limit: 0.5 log TCID_50_/mL) after 3 min of treatment.

The benzalkonium chloride-based product diluted to a concentration of 0.066% showed log reduction values of 3.6, 3.7, 4.0, and 4.4 for 0.5, 1, 2, and 3 min, and no CPE was observed (detection limit: 0.5 log TCID_50_/mL) after 5 min of treatment.

### Evaluation of the efficacy of the formulated hand sanitizers against the HCoV-229E suspension

**Fig. 2:**
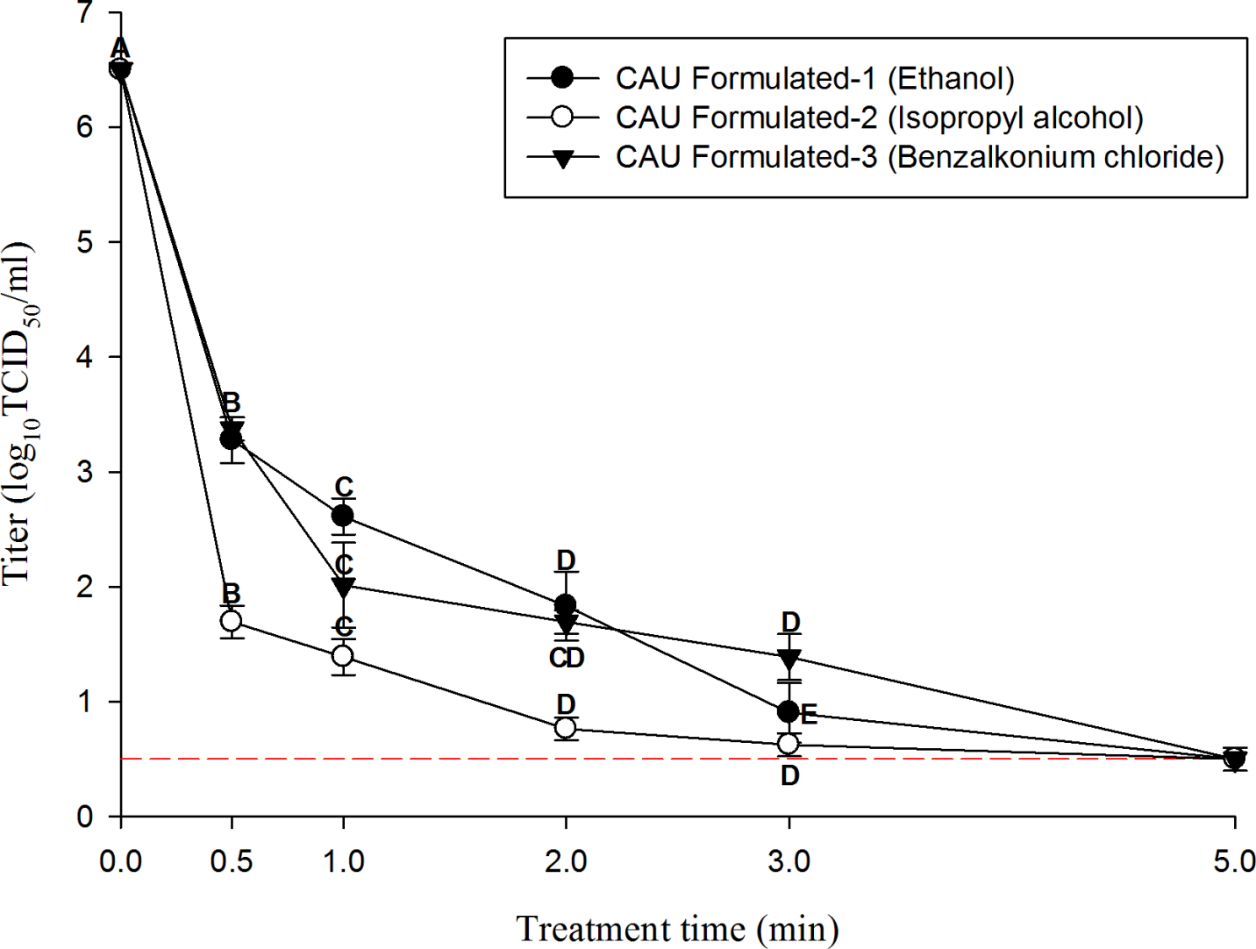
Inactivation of HCoV-229E in suspension by formulated hand-sanitizers. The error bar represents standard deviations of the means (SD). A-E indicate significant differences (p < 0.05) of each hand-sanitizers by treatment time. The short dash line informs the detection limit (0.5 log TCID_50_/ml)

Figure 2 shows the efficacy of the formulated ethanol-, isopropyl alcohol-, and benzalkonium chloride-based hand sanitizers against HCoV-229E in suspension. The initial titer of HCoV-229E was 6.5 log TCID_50_/mL. The formulated 70% ethanol hand sanitizer showed HCoV-229E log reduction values of 3.2, 3.8, 4.6, and 5.5 log TCID_50_/mL for 0.5, 1, 2, and 3 min and no CPE (detection limit: 0.5 log TCID_50_/mL) was observed after 5 min of treatment, respectively. The 70% isopropyl alcohol product exhibited log reduction values of 4.8, 5.1, 5.7, and 5.8 for 0.5, 1, 2, and 3 min, and no CPE (detection limit: 0.5 log TCID_50_/mL) was observed after 5 min of treatment.

Formulated benzalkonium chloride products diluted to a concentration of 0.066% showed log reduction values of 3.1, 4.4, 4.8, and 5.1 for 0.5, 1, 2, and 3 min, and the result of the 5 min treatment group were the same as those of the above two disinfectants. (detection limit: 0.5 log TCID_50_/mL)

### Effect of caffeic acid and vanillin on the viability of MRC-5 cells by MTT assay

**Fig. 3:**
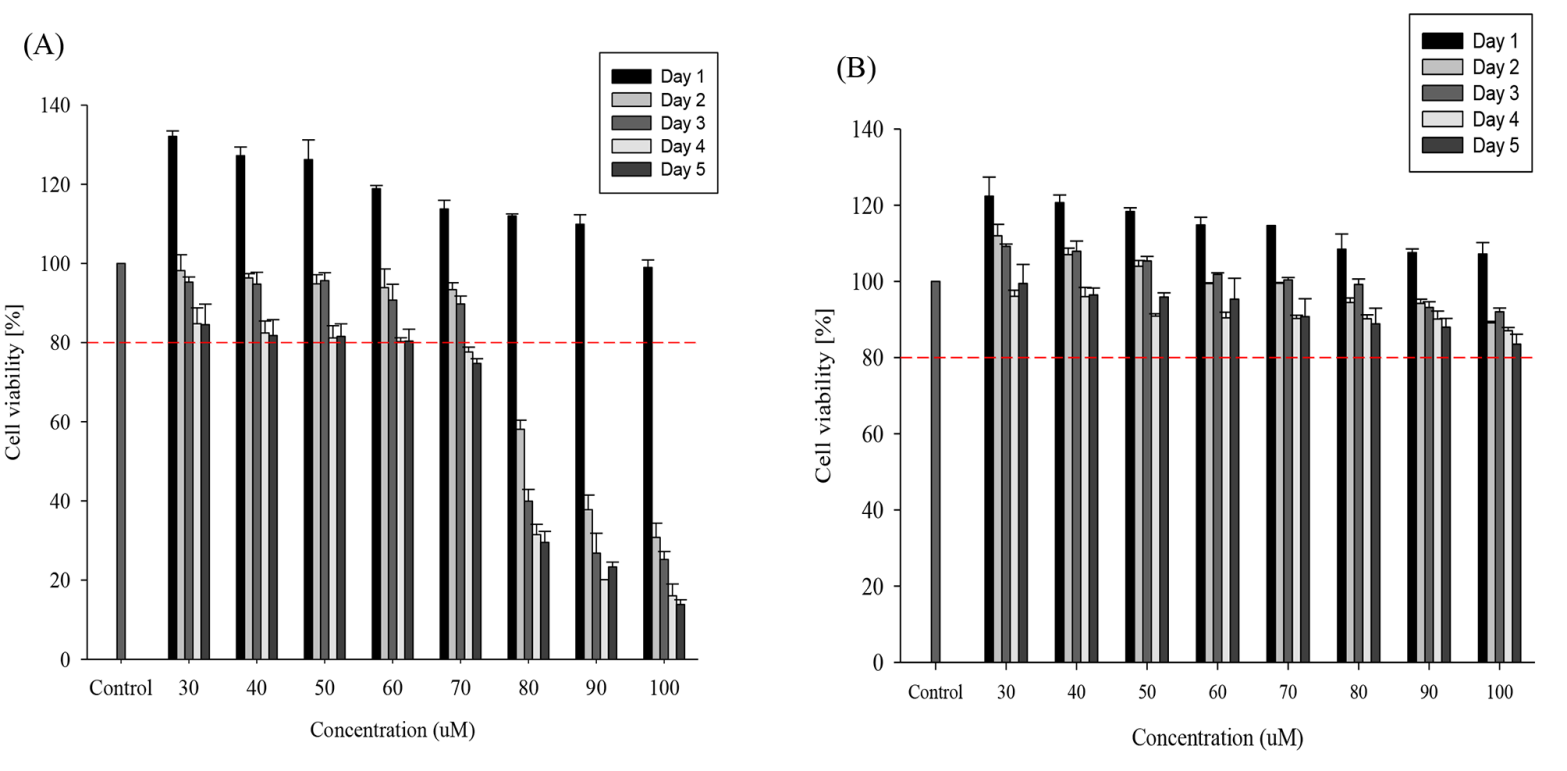
Cytotoxicity of MRC-5 in various concentration by caffeic acid **(A)** and vanillin **(B)** The short dash line informs the 80% of cell viability in each caffeic acid and vanillin concentration

MTT assay was conducted to determine the optimal concentration of caffeic acid and vanillin that can provide antiviral activity without showing cytotoxic effects. Figure 3 shows that caffeic acid and vanillin repress the proliferation of MRC-5 cells at concentrations above 60 µmol and 100 µmol, respectively, for 5 days of treatment. When caffeic acid and vanillin were applied at 60 µmol and 100 µmol for 5 days, the viability of MRC-5 cells remained above 80%.

### Comparison of the efficacy of the formulated hand sanitizers containing natural substances

**Fig. 4:**
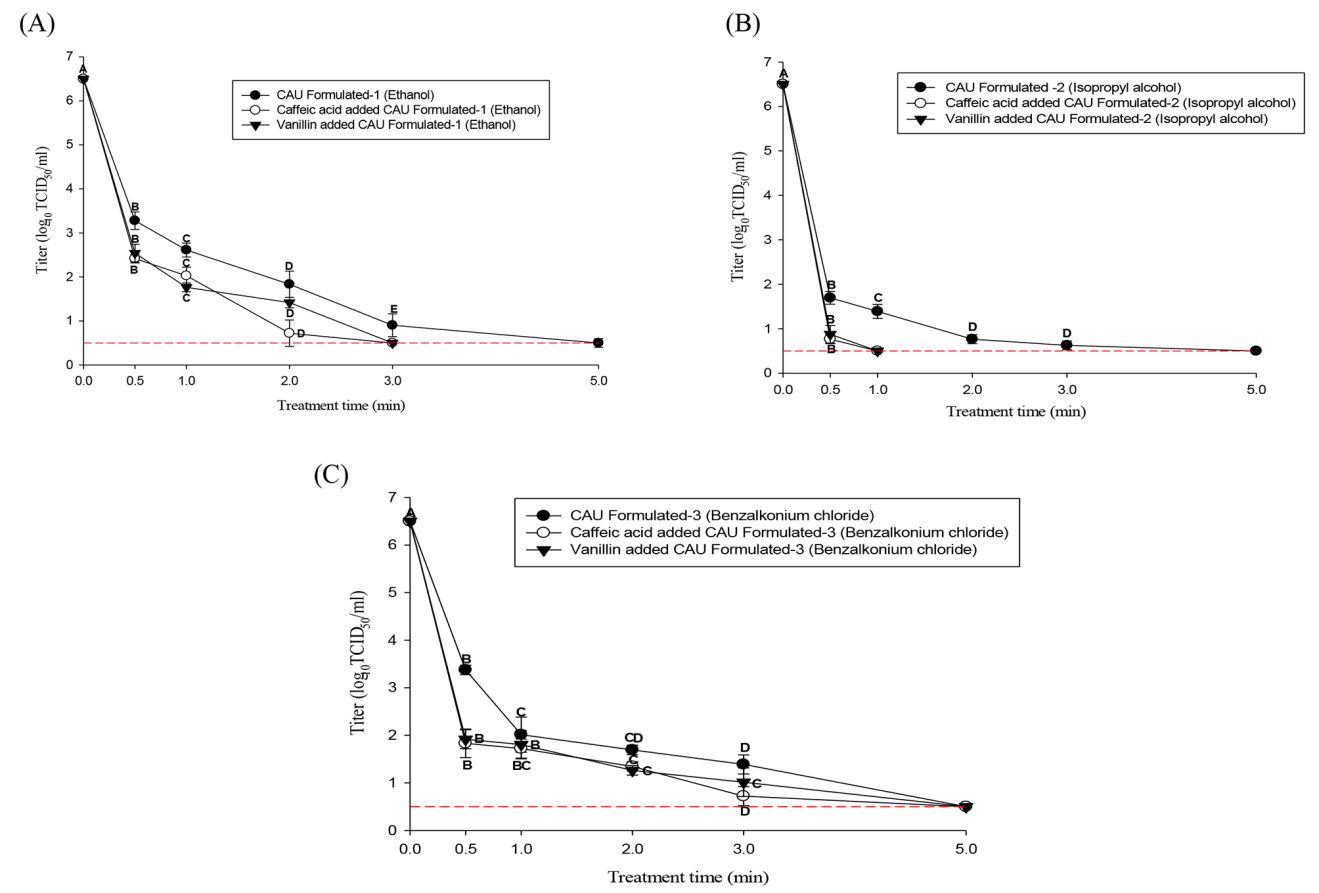
Comparison of the efficacy of the formulated hand sanitizers containing natural substances against suspension. **(A)**, **(B)** and **(C)** represent ethanol, isopropyl alcohol and benzalkonium chloride, respectively. The error bars represent standard deviations of the means (SD). A-E indicate significant differences (p < 0.05) by treatment time. The dashed line indicates the detection limit (0.5 log TCID_50_/mL)

Figure 4 A, B and C shows the efficacy of natural substances added to formulated hand sanitizers against HCoV-229E in suspension. The initial titer of HCoV-229E was 6.5 log TCID_50_/mL.

The formulated 70% ethanol hand sanitizer showed HCoV-229E log reduction values of 3.2, 3.8, 4.6, and 5.5 log TCID_50_/mL for 0.5, 1, 2, and 3 min and no CPE (detection limit: 0.5 log TCID_50_/mL) was observed at 5 min of treatment. The sanitizer made with 70% ethanol and 60 µmol of caffeic acid showed HCoV-229E log reduction values of 4.0, 4.4, and 5.7 for 0.5, 1, and 2 min of treatment, respectively, and no CPE (detection limit: 0.5 log TCID_50_/mL) was observed after 3 min of treatment. The formulated 70% ethanol hand sanitizer with 100 µmol of vanillin showed HCoV-229E log reduction values of 3.9, 4.7, and 5.0 log for 0.5, 1, and 2 min of treatment, respectively, and no CPE (detection limit: 0.5 log TCID_50_/mL) was observed after 3 min of treatment. The formulated 70% isopropyl alcohol product showed log reduction values of 4.8, 5.1, 5.7, and 5.8 for 0.5, 1, 2, and 3 min, and no CPE (detection limit: 0.5 log TCID_50_/mL) was observed for 5 min treatment. The product formulated with 70% isopropyl alcohol and 60 µmol of caffeic acid showed 5.7 log reduction for 0.5 min of treatment, and no CPE (detection limit: 0.5 log TCID_50_/mL) was observed after 1 min of treatment. The product formulated with 70% isopropyl alcohol and 100 µmol of vanillin showed a log reduction value of 5.6 for 0.5 min of treatment, and no CPE (detection limit: 0.5 log TCID_50_/mL) was observed after 1 min of treatment.

The benzalkonium chloride sanitizer diluted to 0.066% showed log reduction values of 3.1, 4.4, 4.8, and 5.1 for 0.5, 1, 2, and 3 min of treatment. The product formulated with 0.066% benzalkonium chloride and 60 µmol of caffeic acid showed log reduction values of 4.6, 4.7, 5.1, and 5.7 for 0.5, 1, 2, and 3 min of treatment, respectively. The product formulated with 0.066% benzalkonium chloride and 100 µmol of vanillin exhibited log reduction values of 4.5, 4.6, 5.2, and 5.4 for 0.5, 1, 2, and 3 min of treatment, respectively.

All formulated benzalkonium chloride-based hand sanitizers containing natural substances showed no CPE (detection limit: 0.5 log TCID_50_/mL) after 5 min of treatment.

### Comparison of the effects of formulated hand sanitizers containing natural substances on porcine skin

**Fig. 5:**
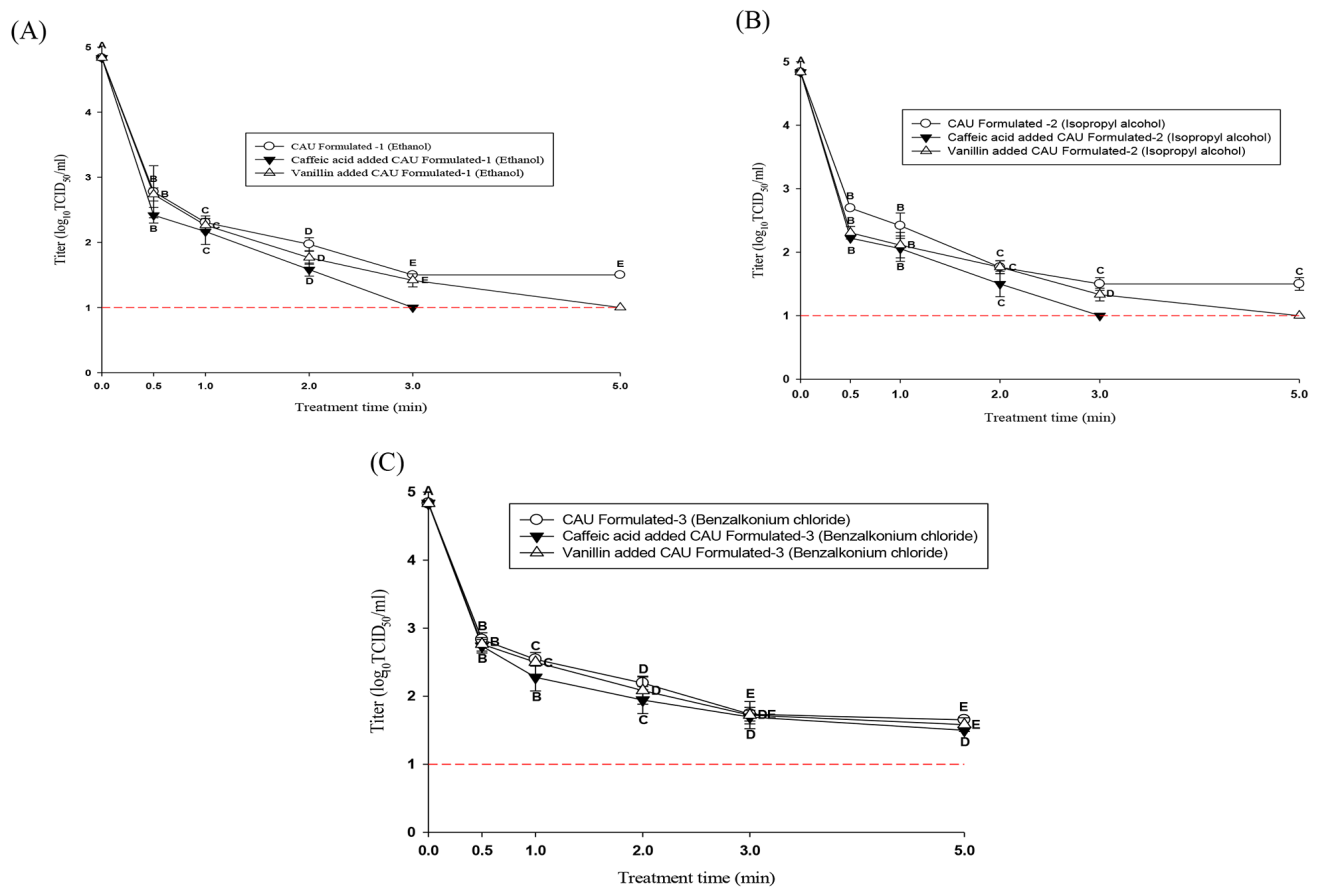
Comparison of the effects by the natural substances in formulated hand sanitizers on porcine skin. **(A)**, **(B)**, **(C)** represent ethanol, isopropyl alcohol and benzalkonium chloride, respectively. The error bars represent standard deviations of the means (SD). A-E indicate significant differences (p < 0.05) by treatment time. The dashed line indicates the detection limit (1.0 log TCID_50_/mL)

Figure 5 A, B and C shows the efficacy of formulated hand sanitizers containing natural substances against HCoV-229E on porcine skin.

The initial titer of HCoV-229E was 7.0 log TCID_50_/mL, and 4.8 log TCID_50_/mL was recovered. The formulated 70% ethanol product showed HCoV-229E log reduction values of 2.0, 2.5 and 2.8 for 0.5, 1, and 2 min of treatment, respectively, and a 3.3 log reduction was observed after 3 min of treatment. The product formulated with 70% ethanol and 60 µmol of caffeic acid showed log reduction values of 2.4, 2.6, and 3.2 for 0.5, 1, and 2 min of treatment, respectively, and no CPE (detection limit: 1.0 log TCID_50_/mL) was observed after 3 min of treatment. The sanitizer formulated with 70% ethanol and 100 µmol of vanillin showed log reduction values of 2.1, 2.5, 3.0 and 3.4 for 0.5, 1, 2, and 3 min of treatment, respectively, and no CPE (detection limit: 1.0 log TCID_50_/mL) was observed after 5 min of treatment.

The formulated 70% isopropyl alcohol product showed HCoV-229E log reduction values of 2.1, 2.4 and 3.0 for 0.5, 1, and 2 min of treatment, respectively, and a 3.3 log reduction was observed after 3 min of treatment (detection limit: 1.0 log TCID_50_/mL). The sanitizer formulated with 70% isopropyl alcohol and 60 µmol of caffeic acid showed log reduction values of 2.6, 2.7 and 3.3 for 0.5, 1, and 2 min of treatment, respectively, and no CPE (detection limit: 1.0 log TCID_50_/mL) was observed after 3 min of treatment. The sanitizer formulated with 70% isopropyl alcohol and 100 µmol of vanillin showed log reduction values of 2.5, 2.7, 3.0 and 3.5 for 0.5, 1, 2, and 3 min of treatment, respectively, and no CPE (detection limit: 1.0 log TCID_50_/mL) was observed after 5 min of treatment.

The 0.066% benzalkonium chloride product showed log reduction values on porcine skin of 2.0, 2.2, 2.6, 3.1 and 3.1 for 0.5, 1, 2, 3, and 5 min of treatment, respectively (detection limit: 1.0 log TCID_50_/mL). The 0.066% benzalkonium chloride-based sanitizer with 60 µmol of caffeic acid showed log reduction values of 2.1, 2.5, 2.8, 3.1 and 3.3 for 0.5, 1, 2, 3, and 5 min of treatment, respectively (detection limit: 1.0 log TCID_50_/mL). The 0.066% benzalkonium chloride-based sanitizer with 100 µmol of vanillin showed log reduction values of 2.0, 2.3, 2.7, 3.1 and 3.2 for 0.5, 1, 2, 3, and 5 min of treatment, respectively (detection limit: 1.0 log TCID_50_/mL).

### Verification of the coated films by ATR-FTIR analysis

**Fig. 6:**
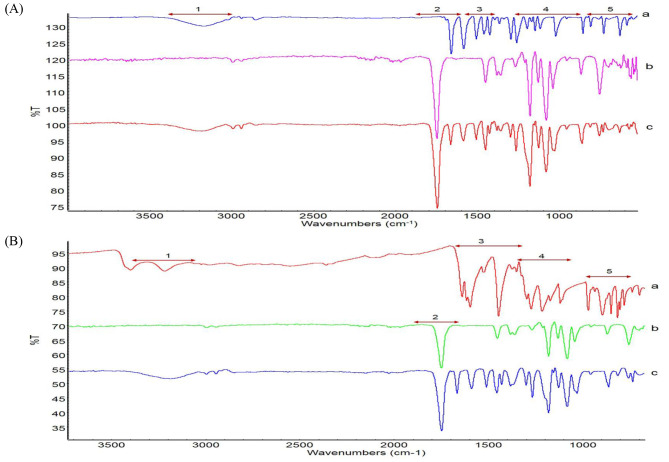
ATR-FTIR analysis of the **(A)** vanillin-coated PLA films and **(B)** caffeic acid-coated films. Each alphabet represents **(a)** Pure vanillin and Pure caffeic acid, **(b)** PLA film, and **(c)** vanillin-coated PLA film and caffeic acid PLA film. Different numbers (1?5) indicate the hydroxyl, carboxyl, conjugated interaction, phenolic, amorphous region, and phenolic/carboxylic functional groups

The PLA films coated with vanillin (VP) and caffeic acid (CP) were analyzed by ATR-FTIR and compared with the pure substances. A verified reference library reported by previous studies was used for data comparison and the data are summarized in Table 2. PLA exhibited three distinct functional groups, identified as carboxylic, phenolic, and amorphous crystalline regions in pure form, located at 1748.7 and 1180.4, 1130–1041.3, and 868.6–753.9 cm^− 1^ (Fig. 6A). Following vanillin coating on the PLA surface, the identical carboxylic group of PLA was found at 1747.93 cm^− 1^. The –C = O stretching bonds were observed in the coated film, which exhibited similar spectra to that of pure vanillin. Moreover, conjugated interactions were observed at 1586–1509.4 cm^− 1^. However, the pure form of vanillin was not detected on the coated surface. In addition, hydroxyl stretching, and related deformations were detected in the same position as in the vanillin spectrum (3186.2 cm^− 1^) (Fig. 6A). After coating PLA with caffeic acid, similar carboxyl groups as those of PLA were detected (Table 2; Fig. 6B). Conjugated interactions were found at 1590.1, 1454.3, 1429.4, 1298.8, 1265.5, and 1126.7 cm^− 1^. Similar to PLA, carboxylic (1747.7 cm^− 1^) and phenolic groups (1180.2 cm^− 1^) were also detected. Furthermore, hydroxyl stretching, and phenolic/carboxylic deformations were the same as in pure caffeic acid (3185.5 and 860.7 cm^− 1^, respectively) (Fig. 6B).

**Table 2:** Spectral assignments and major bands recorded for the various films analyzed by ATR-FTIR.

Wavenumbers (cm^− 1^)					Spectral assignments*	Functional group	Reference
PLA	Vanillin	Vanillin-PLA	Caffeic acid	Caffeic acid-PLA			
-	3180.4	3186.2	3219.8	3185.5	ν(O-H)	Hydroxyl	[43] [44]
1748.7		1747.93		1747.7	ν(C = O)	Carboxylic	[45]
	1662.3	1665.6		1665.7	ν(C = O)	Carboxylic	[46]
			1640–1597.3	1590.1	ν(C = O)	Carboxylic	[47]
	1586.1	1588.3			ν(C-C-C) with –(C = O)	Conjugated interaction	[46]
	1508.6	1509.4			ν(C-C-C)	Benzene ring vibrations	[44]
			1446.6	1454.3 1429.4	ν(C-C) with δ(C-C-H)	Carboxylic	[47]
			1295.5	1298.8	ν(C-O-H)	Phenolic	[47]
			1272.1	1265.5	ν(O-H)	Phenolic	[47]
1180.4		1180.5		1180.2	ν(C-O) with –(CH-O)	Phenolic	[45]
			1171–1118.1	1126.7	ν(C-H)	Intensive stretching vibrations	[47]
	1150.84				ν(C-O-C)	Pure vanillin	[44]
1130.8 1081.4 1041.3		1127.7 1081.5 1030.3		1081.3 1029.8	ν(C-O) with –(COO^−^)	Carboxylic	[45]
			894.3	860.7	δ(C-O-H)	Phenolic or carboxylic	[48]
868.6–753.9		755.9 733.4			-	Amorphous region	[44]

### Comparison of the efficacy of PLA and natural substances added to PLA films against the HCoV-229E suspension

**Fig. 7:**
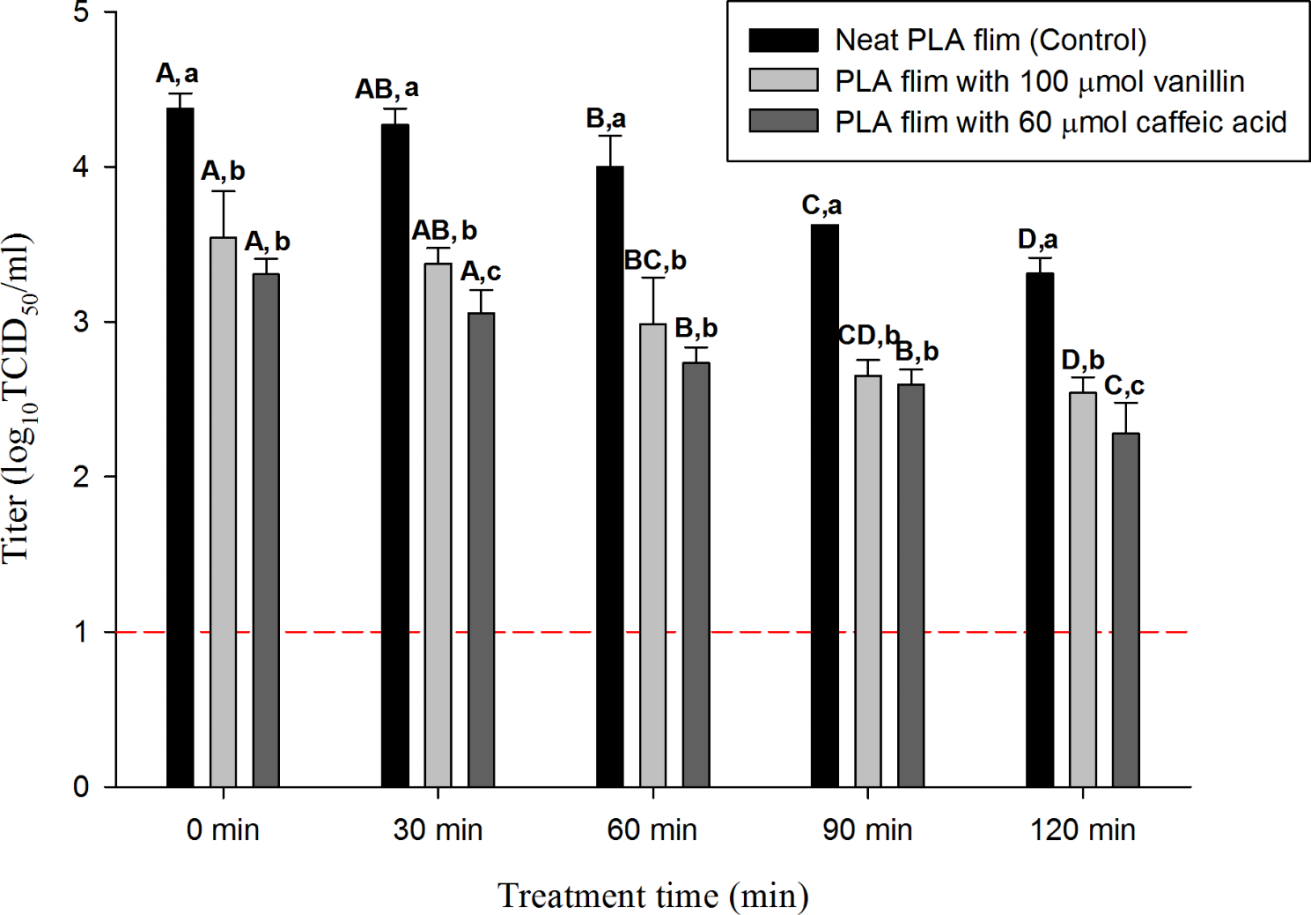
Comparison of the efficacy of neat PLA (NP) and natural substances (CP, VP) added PLA films against suspension.The error bar represents standard deviation of the means (SD). A-D indicate significant differences (p<0.05) of each antiviral films by treatment time. a-c indicates significant difference between NP and VP, CP in each treatment time group. (p<0.05) The short dash line informs the detection limit (1.0 log TCID_50_/ml)

Figure 7 shows the efficacy of PLA and natural substances added to PLA films against the HCoV-229E suspension. The initial titer of HCoV-229E was 5.3 log TCID_50_/mL, and 4.3, 3.3 and 3.5 log TCID_50_/mL were recovered at 0 min after the drying process on each PLA film.

In the neat PLA film (NP), the titer of HCoV-229E was 4.2, 4.0, 3.6 and 3.3 log for 30, 60, 90, and 120 min of treatment, respectively (detection limit: 1.0 log TCID_50_/mL). Compared with the NP as a control group, the CP showed log reduction values of 1.2, 1.2, 1.0 and 1.0 log for 30, 60, 90, and 120 min of treatment, respectively (detection limit: 1.0 log TCID_50_/mL).

The VP showed log reduction values of 0.9, 1.0, 0.9 and 0.7 for 30, 60, 90, and 120 min of treatment, respectively (detection limit: 1.0 log TCID_50_/mL).

### Evaluation of the efficacy of the neat PLA film and natural substance added PLA film according to the number of contacts with porcine skin

Porcine skin was used as a surrogate model for human skin to investigate the antiviral properties of the PLA film depending on the presence of natural substances and the number of contacts between the film and skin. In addition, the conditions of friction and virus loss due to surface contact frequency were considered.

**Fig. 8:**
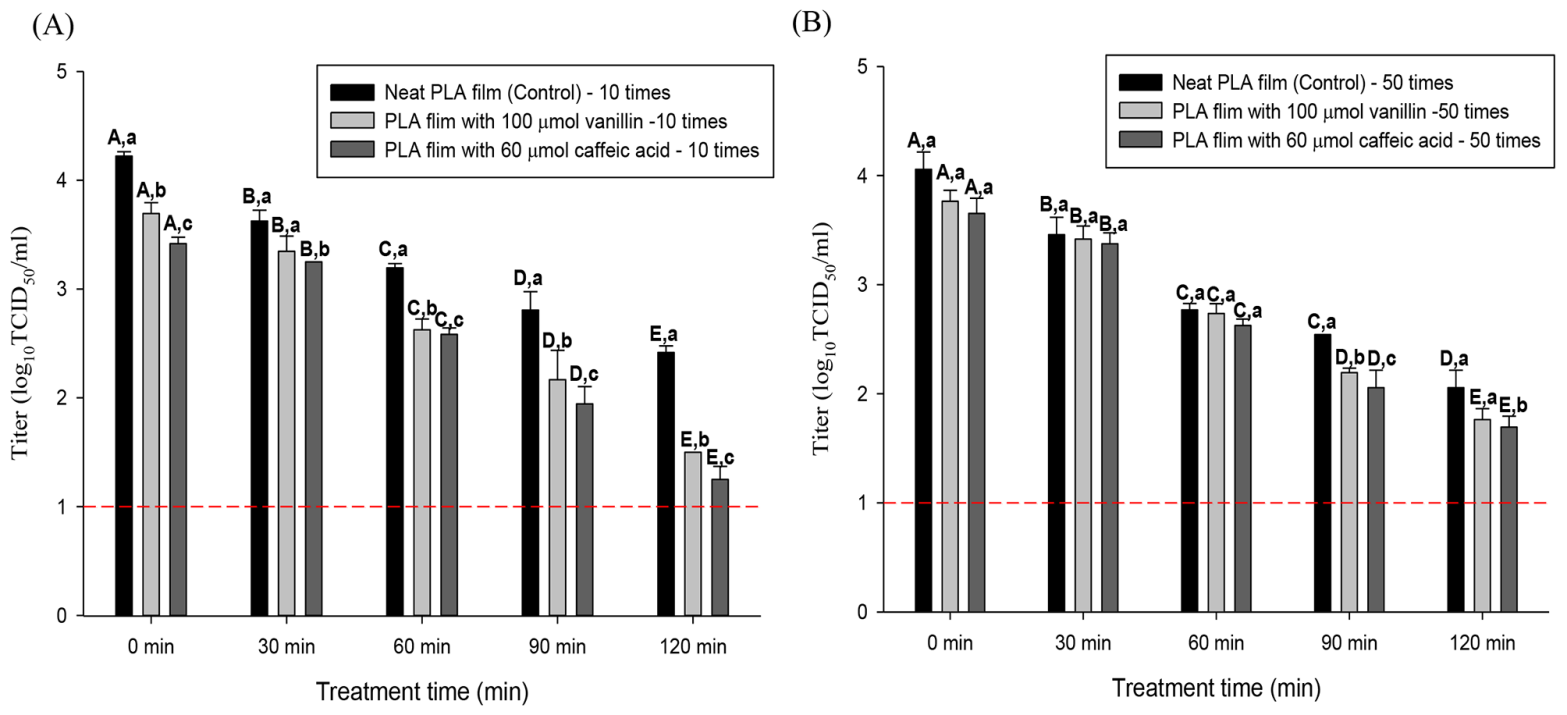
Comparison of antiviral effects according to the number of contacts on porcine skin. **(A)** and **(B)** represent the efficiency of neat, caffeic acid and vanillin added PLA film by contact times on porcine skin, respectively. The error bars represent standard deviations of the means (SD). A-E indicate significant differences (p<0.05) of each antiviral films by treatment time. a-c indicates significant difference between NP and CP, VP in each treatment time group. (p<0.05) The short dash line informs the detection limit (1.0 log TCID_50_/ml)

The results of friction and virus loss due to 10 times of surface contact are shown in Fig. 8A. In the neat PLA film, the viral titer decreased as the number of rubs and time increased. The initial viral titer was 6.1 log TCID_50_/mL and, at 0 min, 4.2 log TCID_50_/mL of the virus were recovered after 10 times rubs. After 10 rubs on porcine skin, the viral titers showed values of 3.6, 3.1, 2.8 and 2.4 log for 30, 60, 90, and 120 min of treatment, respectively.

Antiviral films with natural substances showed a significant differences in efficiency compared to most of NP group and the treatment time groups when contacted 10 times. In CP, the initial viral titer was 6.1 log TCID_50_/mL and, at 0 min, 3.4 log TCID_50_/mL of the virus were recovered after 10 rubs. The efficacy of the prepared films was compared to that of the NP as a control. When each sample was rubbed 10 times with the CP, the viral titer showed log reductions of 0.3, 0.6, 0.8 and 1.1 for 30, 60, 90, and 120 min of treatment, respectively. Likewise, VP showed the same antiviral tendency as CP. The initial viral titer was 6.1 log TCID_50_/mL and, at 0 min, 3.7 log TCID_50_/mL of the virus were recovered after 10 rubs. The efficacy of the prepared films was compared to NP as a control. When each sample was rubbed 10 times with the VP, log reductions were 0.2, 0.5, 0.6 and 0.9 for 30, 60, 90, and 120 min of treatment, respectively.

However, compared to the 10 times rubbed groups, the antiviral PLA films containing natural substances showed relatively low effect on the viral titer in 50 times rubbed groups. The efficacy of PLA films containing 50 times rubs against the HCoV-229E suspension is shown in Fig. 8B. In the NP, initial viral titer was 6.1 log TCID_50_/mL and, at 0 min, 4.0 log TCID_50_/mL of the virus were recovered after 50 rubs. After 50 rubs on porcine skin, the viral titers showed values of 3.4, 2.7, 2.5 and 2.0 log for 30, 60, 90, and 120 min of treatment, respectively. In CP and VP, the initial viral titer was 6.1 log TCID_50_/mL and, at 0 min, and 3.6 log TCID_50_/mL of the virus were recovered after 50 rubs. The efficacy of the prepared films was compared to NP as a control. When each sample was rubbed and 50 times with the CP and VP, the viral titer showed log reductions of 0.08, 0.1, 0.4 and 0.3 and 0.04, 0.03, 0.3 and 0.2 for 30, 60, 90, and 120 min of treatment, respectively.

## Discussion

Commercial hand sanitizers based on ethanol, isopropyl alcohol, and benzalkonium chloride were selected by their sales ranking in Korea. All hand sanitizers had to meet the European Norm (EN) 14,476 performance standards of 4 log reduction within 2 min [49]. Thus, the commercial sanitizers used in this study were evaluated by applying the EN 14,476 protocol to quantify the log reduction value in suspension. The results obtained showed values above 4 log within 2 min. These results agree with inactivation experiments against SARS-CoV-2, using alcohol-based commercial sanitizers, which have shown values above 3 log within 30 s. Furthermore, commercial isopropyl- and benzalkonium chloride-based products have shown similar results against SARS-CoV-2 (reductions above 3 log within 30 s) [13, 50]. The ethanol, isopropyl alcohol, and benzalkonium chloride hand sanitizers formulated in this study were also evaluated by applying the EN 14,476 protocol. These products reduced the viral titer by more than 4 log within 2 min. In particular, isopropyl alcohol showed greater efficacy in reducing the viral titer than ethanol and benzalkonium chloride in the 30-s treatment groups (titer reduced by 1.5 and 1.6 log, respectively). This agrees with a previous study [51] that mentioned that isopropyl alcohol has one more carbon group than ethanol in its molecular formula, which contributes to the inactivation of the lipophilic and enveloped structures of SARS-CoV-2.

As hand sanitizers are used to clean up the skin of the hands, porcine skin was used as a surrogate model of human skin to evaluate the efficacy of the hand sanitizers. Porcine skin is histologically similar to human skin and has been used in several studies to evaluate the efficacy of antimicrobial agents. Therefore, porcine skin is expected to yield similar results to human skin. In a previous study [52] which evaluated the efficacy of disinfectants on porcine skin, an ethanol-based skin and wound cleanser (AWC2) showed 3 log reduction for 5 min of treatment after 6 log PFU/ml of SARS-CoV-2 inoculated. Likewise, the present study showed a decrease of 3 log when the skin was treated for 3 min. Nevertheless, even after 10 min of treatment with the AWC2 disinfectant, the decrease was lower than 0.5 log [52]. In addition, no change in the reduction value was observed after 3 min in the present study. These results may be due to the use of the disinfectant in the presence of organic matter like serum or protein, which hinders the antiviral effect [53]. Therefore, organic substances present in the porcine skin significantly decreased (*p* > 0.05) the disinfection power, which may explain why the antiviral effect of the hand sanitizers formulated in this study stopped after 3 min.

Several investigations focused on the significant antiviral properties of natural compounds which have mainly plant’s fragrant and biological properties. They are complex mixtures of lipophilic and volatile secondary metabolites. There are several groups of plant antimicrobials including phenolic compounds, saponins, thiosulfinates, glucosinolates, terpenoids and isoflavonoids [54]. The majority of antiviral studies has been focused on enveloped viruses while limited research has been conducted on the efficacy of these natural compounds against non-enveloped viruses. Therefore, little is known regarding to antiviral activities of natural compound such as essential oils and plant extracts against non-enveloped viruses. However, non-enveloped viruses are known to be more resistant to environmental conditions and the action of antimicrobials than enveloped viruses [55]. Various natural compounds can inactivate the virus by interfering with the virion envelope structure of the enveloped virus or by adsorption to the host cell, whereas the protein capsid of the non-enveloped virus protects the nucleic acid of the virus and prevents the adsorption of the virus to the host cell. Interfering with entry can reduce the efficiency of virus inactivation [56]. found that the application of essential oils was not effectively inactivate murine norovirus and human adenovirus which are both non-enveloped viruses and concluded that Essential oils are not alternatives to reduce or eliminate non-enveloped viruses in the food industry. More recently, [57] investigated the antiviral activity of plant-derived products including black chokeberry, elderberry, and pomegranate juice, as well as green tea against surrogate-modified vaccinia virus Ankara, and SARS-CoV-2, influenza A virus (IAV), and adenovirus Type 5. Although their antiviral efficiency was vary as the composition of each natural compounds were different, however, the tested natural compounds were reduced the most of viruses except adenovirus Type 5 which is non- enveloped virus which was less susceptible to the tested natural compounds However, some antiviral effects also have been found on small enteric viruses such as human norovirus, murine norovirus-1, rotavirus, and adenovirus have been found to have some effect on the virus by acting to some extent on the protein capsid of these viruses [58].

Although no research has been conducted to evaluate the efficacy of hand sanitizers with caffeic acid and vanillin, an experiment was conducted to determine the synergistic effect of natural substances and the hand sanitizers made with them against HCoV-229E. As a result, hand sanitizers containing caffeic acid and vanillin showed a viral titer that was reduced to the detection limit, even in the porcine skin experiments, and the time to reach the detection limit was shortened for the viral suspension. In the present study, the time to reach the detection limit of the virus titer in suspension was shortened from 5 to 3 min for ethanol and 5 min to 1 min for isopropyl alcohol after the natural substances were added to the hand sanitizers. Furthermore, when hand sanitizers with natural substances were applied to the porcine skin, ethanol and isopropyl alcohol products containing caffeic acid inactivated HCoV-229E in 3 min. Products containing vanillin completely inactivated the virus in 5 min. By contrast, benzalkonium chloride exhibited no changes in the time to reach the detection limit of the virus titer, even if natural substances were added. Previous reports [59] and [60] have mentioned that benzalkonium chloride showed lower efficacy compared to alcohol-based agents against SARS-CoV-2 and was more sensitive in the presence of organic matter compared to other disinfectants. However, the present study showed sufficient antiviral effects for this sanitizer as the viral reduction was above 4 log in suspension, which agrees with other reports [61, 62]. Therefore, benzalkonium chloride can also be considered an effective disinfectant against SARS-CoV-2.

Antiviral films, which are some of the ways to prevent the spread of SARS-CoV-2 through the hands, were prepared by mixing PLA and natural substances. FTIR analysis confirmed the conjugation of natural substances and PLA. Vanillin and caffeic acid were readily coupled with the PLA films and displayed equivalent functional linkages during FTIR analysis. Nevertheless, the primary spectrum assignments were identified as the carboxylic and phenolic groups for both vanillin and caffeic acid-coated PLA films. Previous studies [46] and [48] reported similar results. FTIR analysis demonstrated that natural substances such as caffeic acid and vanillin are conjugated with PLA, and chemical or physical conjugation with these natural substances enhances the properties of the PLA films. For example, the addition of caffeic acid to PLA softens the film because the former acts as a plasticizer and may also provide resistance to UV exposure and weathering [63, 64]. Vanillin is dispersed molecularly in an amorphous state within PLA and increases the rate of biodegradation of PLA as well as the modulus and elongation [65, 66]. Since the antiviral film prepared in this study was developed for use in real life as in a previous report [9], efficacy was evaluated by considering two conditions: the extent of the antiviral effects during a short period of time, and the antiviral activity of the natural substances in the presence of organic matter according to the number of contacts. Compared to the neat PLA film (NP), the caffeic acid-added PLA film (CP) and vanillin-added PLA film (VP) showed significant (*p* < 0.05) log viral reductions of 1.0 and 1.2 in 1 h. A previous study [10] argued that droplets containing viruses can be removed through hydrophobic properties (e.g., through bouncing), enhancing the synergistic effect of antiviral substances on hydrophobic surfaces. Therefore, the observed antiviral effects of CP and VP may be due to the mechanism proposed in that study [10]. In addition, the 1 log difference at 0 min between NP and natural substances added films groups (CP, VP) may be the result of the extended (30 min) drying that is likely to affect the films containing natural substances.

Furthermore, to evaluate the antiviral efficacy of natural substances according to the number of contacts with porcine skin, 10 and 50 rubs were designated as intermediate and multiple contacts, based on a reported methodology [9]. The log reduction value of NP decreased as the time and number of rubs increased, which agrees with a previous study [67]. Nevertheless, a difference in the loss rate was detected, which depended on the presence of liquid. Approximately 13–16% of the virus moves to the fomite surface under wet conditions, whereas only about 3–9% moves under dry conditions. In addition, because the friction generated by rubbing can affect viral transfer, the generation of friction during the 50 rubs can result in a decrease in the viral titer [68]. Both CP and VP exhibited a decrease in the viral titer over the processing time. However, as the number of rubs increased (50 times), the effect on viral reduction was relatively low compared to low contact time frequency (10 times). These results can be explained by the factors that affect the antimicrobial activity of natural substances [69]. Phenolic compounds show hindered activity in the presence of nitrogenous compounds or fats and can form complexes with proteins. Thus, films that were rubbed 50 times on porcine skin exhibited a lower antiviral activity than those subjected to 10 rubs because of the presence of organic matter in porcine skin. Nevertheless, in the case of CP and VP, the viral titer almost reached the detection limit (1 log TCID_50_/mL) with values of 1.2 log and 1.5 log after 2 h of treatment. Even if some viral losses occurred due to friction and contact, the films containing caffeic acid and vanillin showed sufficient antiviral activity.

## Conclusion

This study investigated the antiviral efficacy of hand sanitizers and PLA films incorporating natural substances (caffeic acid and vanillin) against HCoV-229E, a surrogate of the SARS-CoV-2 virus. Hand sanitizers sold in the market and those prepared here and incorporating natural substances showed a reduction of more than 4 log within 2 min in suspension. This study also evaluated the efficacy of the prepared hand sanitizers on porcine skin contaminated with HCoV-229E. Overall, the prepared hand sanitizers without natural substances tended to have slightly lower antiviral power than those containing natural substances. Furthermore, when using the latter, the viral titer reached the detection limit. Benzalkonium chloride showed sufficient antiviral activity in suspension and porcine skin; however, after natural substances were incorporated, there was no difference in the time needed to reach the detection limit of the virus. FT-IR analysis of PLA films incorporating caffeic acid and vanillin confirmed that the natural substances and PLA were conjugated. Ten rubs with the films resulted in slightly higher antiviral activity than 50 rubs. Based on the results obtained from this study and literature reports, we suggest removing the organic substances present on the skin with soap and water as much as possible before applying hand sanitizers and antiviral films. In addition, further research is required as various mutations and strains have increased the viability of SARS-CoV-2 on skin and fomites, as well as its resistance to ethanol. Furthermore, new alternatives to prevent infection by SARS-CoV-2 should take advantage of the synergistic effect of sanitizers with phytochemicals or other antiviral substances.

## Data Availability

Not applicable. All relevant data are within the paper.

## List of abbreviations

COVID-19: Coronavirus disease 2019
SARS-CoV-2: Severe Acute Respiratory Syndrome Coronavirus 2
HCoV-229E: Human-infecting Coronavirus 229E
PLA: Poly Lactic Acid
ATR FT-IR: Attenuated total reflectance-Fourier transform infrared spectroscopy analysis
CPE: Cytopathic effect
NP: Neat PLA film
CP: Caffeic acid-added PLA film
VP: Vanillin-added PLA film
